# Recycling Foundry Sands in Concrete: A Comparative Study on the Use of Green Sand and Chemically Bonded Sand as Partial Replacements for Natural Sand

**DOI:** 10.3390/ma18184245

**Published:** 2025-09-10

**Authors:** Pietro Di Maida, Corrado Sciancalepore, Enrico Radi, Luca Lanzoni, Daniel Milanese

**Affiliations:** 1 Dipartimento di Scienze e Metodi dell’Ingegneria, University of Modena and Reggio Emilia, 41122 Modena, Italy; pietro.dimaida@unimore.it; 2 Dipartimento di Ingegneria dei Sistemi e delle Tecnologie Industriali, University of Parma, 43124 Parma, Italy; corrado.sciancalepore@unipr.it (C.S.); daniel.milanese@unipr.it (D.M.); 3 Dipartimento di Ingegneria “Enzo Ferrari”, University of Modena and Reggio Emilia, 41125 Modena, Italy; luca.lanzoni@unimore.it

**Keywords:** concrete, recycling, green sand, chemically bonded sand, strength properties, durability

## Abstract

Currently, many foundries successfully reuse sand multiple times within their production cycle. However, when the sand can no longer be reused, it is disposed of, resulting in environmental damage and high disposal costs for the company. The present research aims to explore the potential reuse of foundry sands as fine aggregate in concrete. Since this by-product is classified as non-hazardous waste, it can offer interesting opportunities for the recycling of a material that is currently one of the most widely used in the construction industry. This paper studies the potential reuse of green sand (GS) and chemically bonded sand (CBS) as a partial replacement for natural sand (NS) in concrete. Concrete specimens made with 10%, 20%, and 30% of foundry sand were tested, and a comparative analysis was carried out with the standard mixture in terms of chemical–physical properties, workability, and mechanical properties. The results showed a reduction in the performance of concrete specimens prepared with foundry sands. The lowest reductions in the strength, which were always below 10%, were observed for a 10% inclusion rate of both GS and CBS, with slightly better performance for CBS. Performance reductions tend to increase with higher replacement rates. However, these performance reductions turn out to be acceptable for concrete used in non-structural applications.

## 1. Introduction

Environmental sustainability and the theme of recycling have become increasingly important, especially due to the development and spread of the circular economy. Nowadays, companies are more interested in applying the circular economy model, particularly in response to resource scarcity and the energy crisis.

The foundry industry is divided into sectors depending on the kind of metal produced: ferrous (cast iron and steel) and non-ferrous metal foundries (Aluminum, Lead, Copper, Zinc, Magnesium, Cadmium, and other non-ferrous alloys). In the sand molding system used in most ferrous metal foundries, each mold is single-use, and once the metal is poured, the mold is destroyed to extract the piece. The mold is made of appropriately selected silica sands mixed with binders (clayey or of another type) and/or additives that provide the necessary properties suitable for forming operations. The classification of foundry sand depends on the type of binder used. Two types of binding systems are typically used: clay-bonded systems (green sand—GS) and chemically bonded systems (chemically bonded sand—CBS), which contain thermoset resins [1,2]. Green foundry sand is the most utilized alternative. The typical binder used is bentonite clay. A carbon-based additive is incorporated to enhance heat resistance and improve the quality of the cast surface as well. As a result of this addition, GS takes on a dark hue [1]. Chemically bonded sands are used in core manufacturing, where high strength is required to withstand the heat of molten metal, and in mold manufacturing [3]. Most chemical binder systems consist of an organic binder that is activated by a catalyst, although some systems involve inorganic binders. Numerous analytical checks have been carried out over time by producers and disposal companies, highlighting that foundry sand can be classified as non-hazardous waste [1,3,4,5,6,7]. Therefore, in recent years, foundries have focused on managing waste from production processes by attempting to reduce its quantity, promoting the reuse of materials within their processes, and seeking possible external uses as alternatives to disposal. Several uses of foundry sand have been identified through application experience, including replacing natural aggregates in cement factories, the glass industry, ceramic industry, cement mortars and concrete, brick production, bituminous conglomerates, and road foundations [3,4,5,6,7]. The use of foundry sand as a replacement for raw materials brings environmental benefits and reduces the costs that foundries must bear for disposal. Consequently, a potential reuse of foundry sands would enable companies to make a profit and save costs that are currently high. The construction industry is responsible for 39% of CO_2_ emissions and over 50% of natural resources extracted [1]. Used foundry sand represents the highest amount of solid waste generated by foundries, and it is a significant resource to be reused in various industrial sectors, primarily in construction [8]. Several studies have already been conducted by various researchers to test the incorporation of foundry sands into concrete, although the results are not always consistent, as a high variability in the composition of foundry sands is recorded due to the specific production cycles of individual companies [1,2,3,4,5,6,7]. Certainly, existing studies show that the performance of concrete containing chemically bonded foundry sand differs from that containing green sand [7]. However, the available experimental evidence is insufficient to allow the widespread use of foundry sand in the production of concrete on an industrial scale. The different industrial processes in foundries, the type and chemical composition (e.g., organic content), the amount of chemical binders, and physical characteristics may have different effects on the properties of the resulting concrete, thus requiring further investigation [5]. The study of the potential use of foundry sands in mortars and concrete has been addressed by various researchers worldwide over several years [1,2,3,4,5,6,7]. An analysis of scientific literature reveals multiple results regarding the effect of this material, at different replacement percentages, on the rheological, mechanical, and durability properties of cementitious mixtures, although these results are not always consistent and homogeneous. However, based on existing studies, the following effects on the physical/mechanical properties of concrete can be observed.

Density of hardened concrete

Previous studies show that the use of foundry sands leads to a reduction in the concrete density [1,6], mainly due to two factors: foundry sands generally have lower densities [9,10] compared to natural sand, and their fine particle size leads to a decrease in workability and, ultimately, a greater formation of voids (increased porosity) and reduced compaction [11].

Workability

All studies indicate that the use of foundry sands results in a decrease in the workability of mixtures [1,2,3,4,5,6,7]. This seems to be primarily due to the very fine particle size, which, having a high specific surface area, tends to absorb water from the mixture [12]. Additionally, the presence of clay particles and other impurities also contributes to water loss [13]. The reduction in workability increases with the percentage of foundry sand [1,2,3,4,5,6,7]. For this reason, many studies often rely on the addition of superplasticizers to maintain a suitable water/cement ratio [8,9,12].

Compressive Strength

Regarding compressive strength, there is a discrepancy in results. Some studies suggest that the addition of foundry sand improves compressive strength up to certain percentages, which is justified by the fineness of the sand, leading to a better filling effect and, consequently, greater compactness and density [14,15]. Conversely, other studies indicate that the addition of foundry sand lowers performance at any percentage due to the excessively fine particle size, which reduces workability, requires more water, and results in lower final strength [16,17,18,19]. Furthermore, the presence of clay or resin films on the surface of the particles would hinder the proper formation of hydration products that contribute to the strength of the matrix [1,5]. The performance decrease becomes more pronounced with higher percentages of incorporated foundry sand [1,2,3,4,5,6,7].

Tensile and Flexural Strength

The results in this area are also varied, with some studies demonstrating an increase in strength and others showing a decrease [13,14,15,16,17,18,19]. The decrease is often attributed to the weakening of the interface transition zone (ITZ) between the cement and aggregate due to poor bonding between the sand particles, which is in turn caused by the clay or resin films covering them [1,16,19]. Concrete performance is typically diminished with the incorporation of foundry sand [1,2,3,4,5,6,7].

Water Absorption

The water absorption of the hardened mixtures after a certain number of curing days shows variable results, although with a tendency to increase as the foundry sand content grows [1,2,3,4,5,6,7]. This seems to be due to an increase in porosity caused by a larger formation of voids [11], as a result of the additional water required in the mixture due to the high specific surface area of the particles or reduced compaction due to decreased workability [6].

The variability of all published results seems to largely depend on the different types of foundry sands used, which vary from country to country and even within individual states, among different foundries. However, in most cases, the introduction of foundry sands into cementitious mixtures leads to a reduction in performance [1,6]. Given the variability of foundry sands, it is difficult to derive common behavior and trends. Instead, it seems more practical to focus on specific foundry sands used for similar productions and develop the Best Available Technologies (BAT) that can maximize recycling benefits.

Therefore, several studies [19,20,21,22,23] are still conducted to expand the available experimental data, aiming to enhance the understanding of these by-products and enable their controlled and effective widespread use in the future. The present research is part of this effort.

## 2. Materials and Methods

### 2.1. Reference Concrete

**Figure 1:**
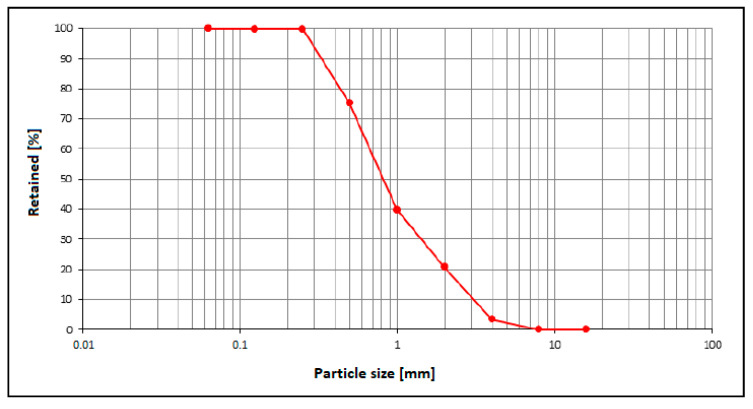
Granulometric distribution of NS.

Mix proportions for the reference concrete are reported in Table 1. The used cement was Portland Cement type CEM II/B-LL 32.5 R according to EN 197-1 [24]. The used water/cement ratio was 0.43. As coarse aggregate, a river gravel was used, consisting of rounded grains with sizes ranging from 4 to 16 mm, having a density of 2.71 g/cm^3^, a water absorption of 0.8%, and compliant with EN 12620 [25] and EN 13242 [26]. As fine aggregate, a river silica sand, hereinafter referred to as NS (Natural Sand), was used, consisting of rounded grains with sizes ranging from 0 to 4 mm (Figure 1), with chemical composition reported in Table 2, having a density of 2.69 g/cm^3^, a water absorption of 0.9%, and compliant with EN 12620.

### 2.2. Foundry Sands

Two different types of foundry sand were used, partially replacing the corresponding natural aggregates. Green sand, hereinafter referred to as GS, originates from a company in northern Italy. The Foundry Division of this company specializes in the production of gray and ductile iron castings in small-to-medium and large series, with unit weights ranging from a few dozen grams up to 7–8 tonnes. The company has an annual production capacity of 2800 tonnes of iron castings. The green sand automatic line, using disposable molds, is highly flexible, accounts for most of the company’s production, and in 2022 generated 1393 tonnes of spent sand, with a disposal cost of 75 €/ton. The green sand, as specified by the company, is produced using 86.5% silica sand, 10% premixed bentonite (consisting of 70% bentonite clay powder formed by silico-aluminates and 30% mineral black), and 3.5% water. After the demolding operation, the sand is sent to a regeneration plant to be reintroduced into the cycle, except for an excess part that is discarded and sent for disposal. The concrete specimens tested in the present research work contain variable fractions of discarded foundry sand. Chemically bonded sand, hereinafter referred to as CBS, comes from the disintegration of cores made by another Italian company. This company operates as a modern aluminum foundry specialized in engine components. Its production process is based on gravity die casting using shell cores made through a thermal hardening shell-molding mechanism, which employs thermosetting resins. In 2022, it generated 1920 tonnes of spent sand, with a disposal cost of approximately €77/ton. The CBS, as specified by the company, is produced using 97.5–98.3% silica sand and 1.7–2.5% partially polymerized and lubricated phenolic resins. For this type of sand, due to the presence of overly coarse elements (reaching sizes of 3–4 cm), a sieving process was carried out, thus eliminating particles with a diameter greater than 4 mm. As the first step, chemical-physical investigations of the samples provided by the company were carried out. To assess the surface morphology and elemental composition, microscopy characterizations were performed using, respectively, an optical digital microscopy Keyence VHX-X1 series (Keyence Italia Spa, Milan, Italy) and an environmental scanning electron microscopy (ESEM) ESEM Quanta-200 (FEI Company, Hillsboro, OR, USA), equipped with the x-ray energy dispersion system (X-EDS) Oxford INCA-350 (Oxford Instruments, Abingdon, Oxfordshire, UK). Physical analyses were then conducted on density, water absorption, and granulometry. The skeletal density of the sands was measured with the helium pycnometer Quantachrome Ultrapyc 1200e (Anton Paar QuantaTec, Graz, Austria). The size distribution curves were obtained using a laser granulometer Mastersizer 300 (Malvern Panalytical, Great Malvern, Worcestershire, UK) with the dry sample dispersion configuration. Water absorption measurement was also carried out in compliance with EN 1097-6 [27].

### 2.3. Casting of the Specimens

For the study of concrete with partial replacement NS with foundry sands, seven mixtures were prepared, as shown in Table 3. The first mixture, ST, is the reference mixture, shown in Table 1. Then, three mixtures were made by replacing 10%, 20%, and 30% by weight of NS with GS. Finally, three further mixtures were made with the same percentages of CBS. The workability of the concrete mixtures was measured using the slump test according to EN 12350-2 [28]. The reference concrete ST fell into a slump consistency class S2. The mixtures containing GS all fell within consistency class S1, with slump values decreasing as the inclusion rate increased, whereas the CBS resulted in consistency class S2, showing a slight reduction in slump compared to the reference concrete. To maintain the same workability class for all the mixtures, a superplasticizer was used. For each mixture, six cubic specimens (150 × 150 × 150 mm) were prepared for compression tests, three cylindrical specimens (100 mm in diameter and 200 mm in height) for splitting tensile strength tests, three prismatic specimens (150 × 150 × 600 mm) for bending tests and three cylindrical specimens (100 mm in diameter and 100 mm in height) for water absorption tests. The concrete was poured into the molds and properly compacted to eliminate voids. After 24 h, the molds were removed, and the specimens were left to cure for 28 days.

## 3. Results

### 3.1. Characterization of Foundry Sands

**Figure 2:**
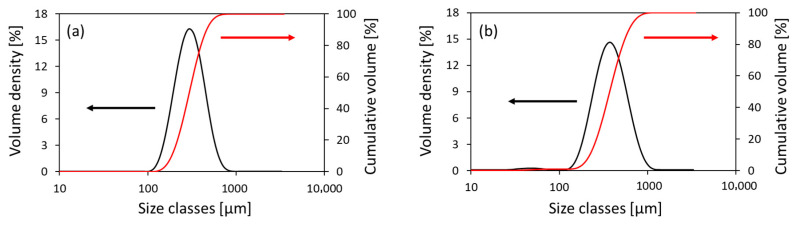
Distributive (black line) and cumulative (red line) distribution curves for CBS (**a**) and GS (**b**) sands.

The densities (d) of GS and CBS were found to be dGS = 2.596 ± 0.006 and dCBS = 2.665 ± 0.007 g/cm^3^ respectively, very similar to the density of natural fine aggregates and coherent with values found in the literature [29,30,31]. GS and CBS grain dimensions fall within the size range expected for fine aggregates, as shown by the distributive and cumulative curves obtained by laser granulometry in Figure 2. For CBS sample, the particle size distribution is characterized by values of D10, D50, and D90 (the percentiles indicating particle sizes less than 10%, 50%, and 90% of the distribution) of 186 ± 7, 297 ± 20, and 464 ± 50 μm, respectively, with an average volumetric mean Dv = 313 ± 24 μm. CBS has a very fine granulometry, with approximately 90% of the grains having a diameter below 0.45 mm, from which a fineness modulus FM = 1.6868 can be derived. The CBS water absorption at 24, 48, and 72 h was found to be null. For the GS sample (Figure 2b), the obtained values were slightly higher (D10 = 203 ± 8, D50 = 348 ± 6, and D90 = 566 ± 12 μm), with a Dv = 367 ± 6 μm and with approximately 90% of the grains having a diameter below 0.57 mm, from which a fineness modulus FM = 1.6864 can be derived. The GS water absorption resulted in 48% at 24 h, 4.5% at 48 h, and 0% at 72 h.

**Figure 3:**
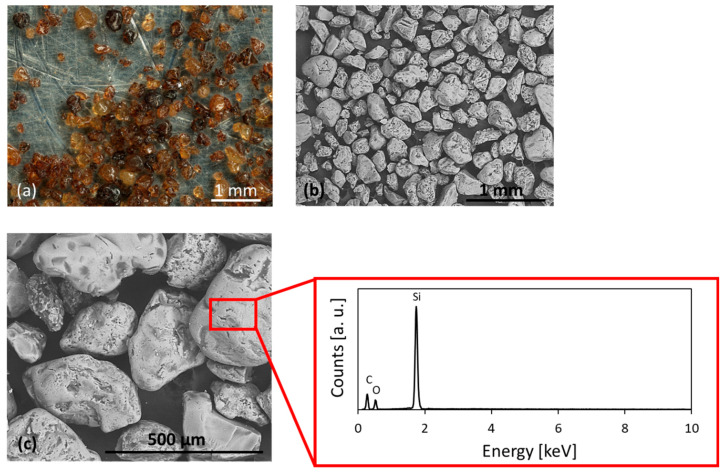
Optical (**a**) and electron (**b**) microscope images for CBS. Detail of the sand (**c**) with corresponding EDS spectrum.

To assess the surface morphology and elemental composition, microscopy characterizations were performed on pristine sands. CBS appears irregularly shaped, slightly rounded (Figure 3a,b). The color is amber and translucent. The surface seems smooth and reflective. According to scale bars in the SEM images, the grain dimensions agree with those found by particle size analysis. Based on the qualitative results of the elemental analysis, CBS consists almost entirely of silicon and oxygen (34 ± 2% and 66 ± 2%, respectively), with small amounts of carbon residues from the thermosetting binder, as shown in the spectrum in Figure 3c. As expected, the detected composition is compatible with that of silica sand, which is mainly composed of SiO_2_.

**Figure 4:**
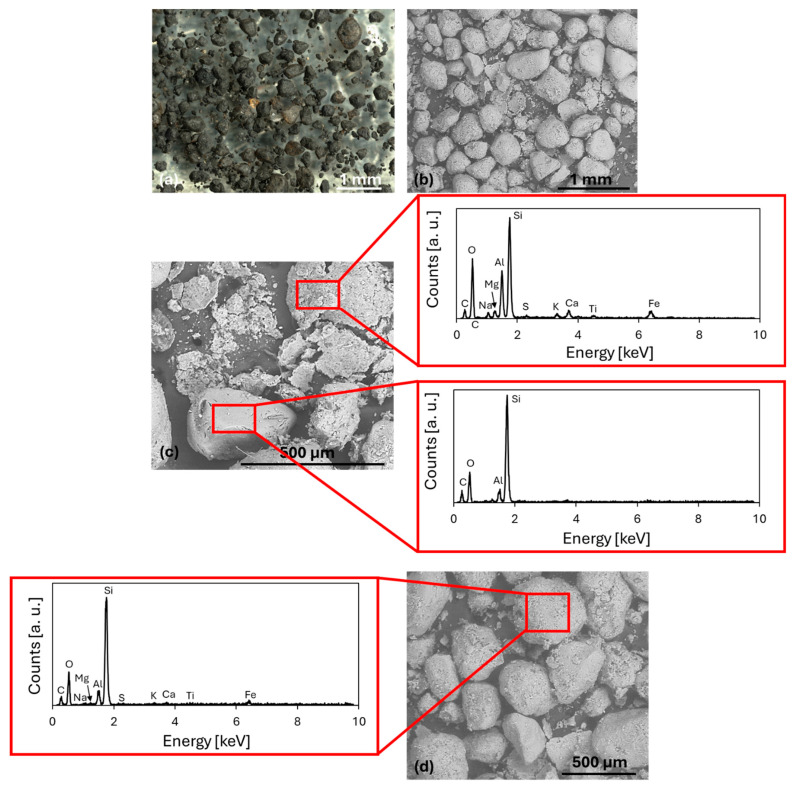
Optical (**a**) and electron (**b**) microscope images for GS; detail of the detached coating and bare sand grain (**c**) with corresponding X-EDS spectra for the outer coating (upper spectrum) and for the grain without coating (lower spectrum); detail of the coated sand (**d**) with corresponding X-EDS spectrum.

In terms of morphology, GS particles appear irregular and roundish (Figure 4a,b). The surface of the particles is rough and matt black in color, typical of bentonite-treated sands [1,2,3,4,5,6]. Bentonite homogeneously coats the surface of the sand grains, and based on experimental evidence obtained from SEM images, its thickness appears to be approximately 10 µm. In areas where the coating has peeled off, the difference with the original grain surface can be observed (Figure 4c).

The elemental composition of the bentonite-based coating is compatible with the typical composition of bentonite [32], while in the uncoated grains the composition is much more similar to that of silica sand, as it appears from the X-EDS spectra in Figure 4c. The elemental composition of GS and bentonite, for comparison purposes, is presented in Table 4, which also includes, for reference, the composition of NS previously reported in Table 2. The GS composition is congruent with the green waste foundry sand [33,34]. Among the elements detected, in the spectrum of GS coated grains (in Figure 4d), carbon is also detected: this element is usually used as an additive to support heat resistance and improve the cast surface [1,2,3,4,5,6].

### 3.2. Density of Hardened Concrete

**Figure 5:**
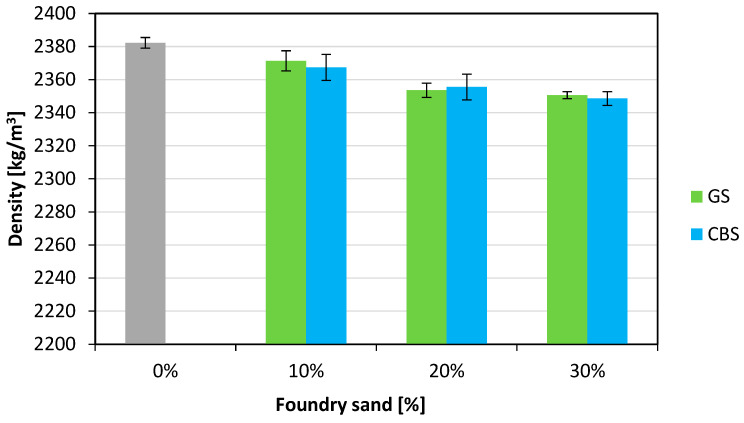
Density of hardened concrete.

Before conducting the mechanical tests for each cube, density measurements of the hardened concrete were taken and are reported in Figure 5 with the respective average values. As observed, as the content of foundry sand increases, a corresponding decrease occurs in the density of hardened concrete. Specifically, the reduction ranges from 0.46% to 1.33% for GS as the percentage of GS increases from 10% to 30%, and from 0.62% to 1.41% for CBS as the percentage of CBS increases from 10% to 30%.

### 3.3. Mechanical Properties

#### 3.3.1. Compressive Strength

**Figure 6:**
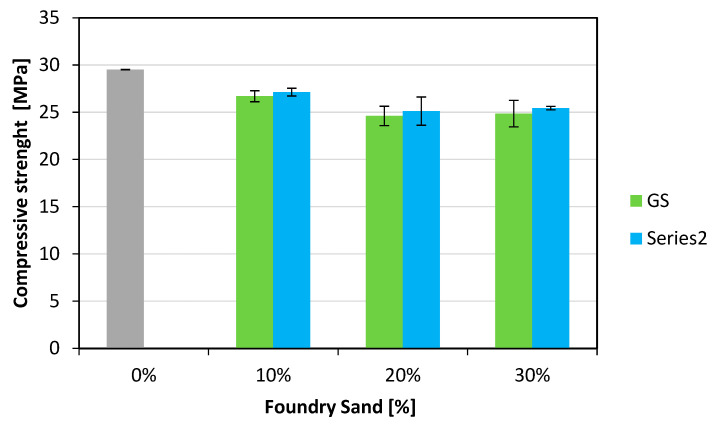
Compressive strength results at 28 days of curing.

Compression tests were carried out on each specimen, measuring 150 *×* 150 × 150 mm, according to the EN 12390-3 [35] standard, by using a 3000 kN capacity compression testing machine (METROCOM Engineering SpA, Novara, Italy), and the results are shown in Figure 6.

**Figure 7:**
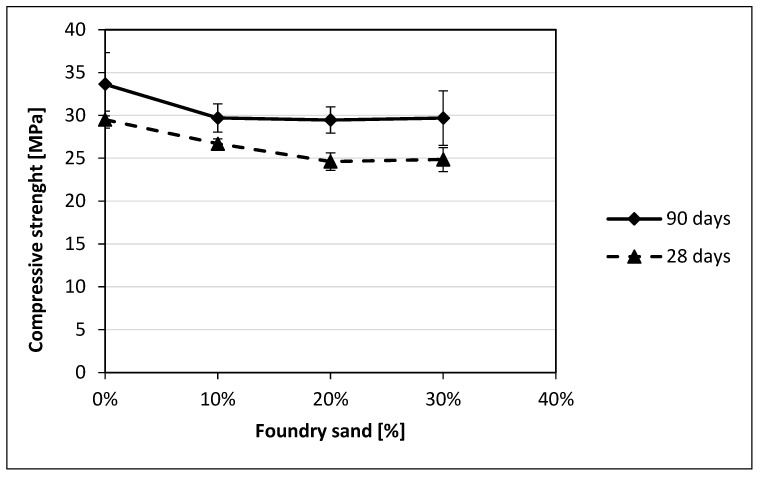
Comparison of compressive strength results at 28 days and 90 days for GS.

**Figure 8:**
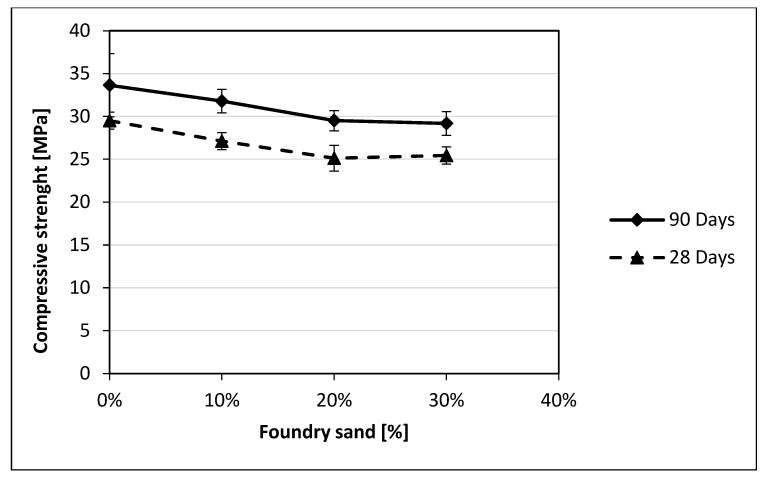
Comparison of compressive strength results at 28 days and 90 days for CBS.

A decrease in the concrete compressive strength is found as the foundry sand content increases. In particular, for GS, a 9% reduction is observed with 10% GS, while a reduction of approximately 16% is observed for 20% and 30% GS contents. On the other hand, smaller reductions are generally observed for CBS, specifically 8%, 15%, and 14% for 10%, 20%, and 30% CBS contents, respectively. A similar behavior was observed for the cubic specimens that were allowed to cure for a period of 90 days to evaluate the effects on long-term compressive strength. Figure 7 and Figure 8 show that the 90-day compressive strengths of both GS and CBS closely follow the same trend observed at 28 days.

#### 3.3.2. Splitting Tensile Strength

**Figure 9:**
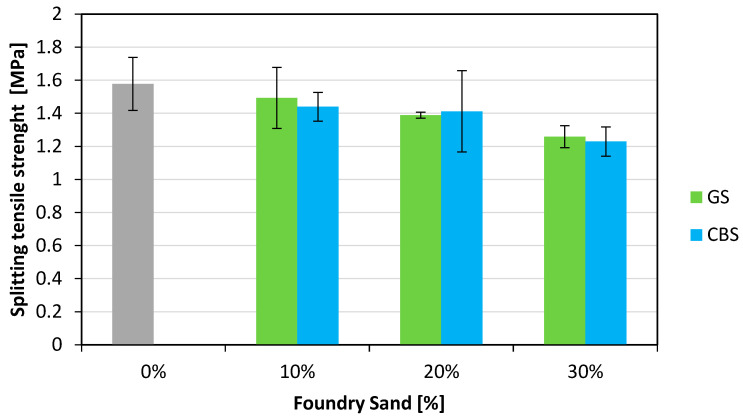
Splitting tensile strength results.

Indirect tensile strength tests were carried out on each cylindrical specimen (length 200 mm and a diameter 100 mm) according to EN 12390-6 [36] standard by using a 3000 kN capacity compression testing machine (METROCOM Engineering SpA, Novara, Italy). The results are shown in the graph in Figure 9. In these tests, a decrease in resistance is also observed with the increasing content of foundry sand. Specifically, for GS, the reductions are 5%, 12%, and 20% for GS contents of 10%, 20%, and 30%, respectively. For CBS, the reductions are 9%, 10%, and 22%.

#### 3.3.3. Flexural Strength

**Figure 10:**
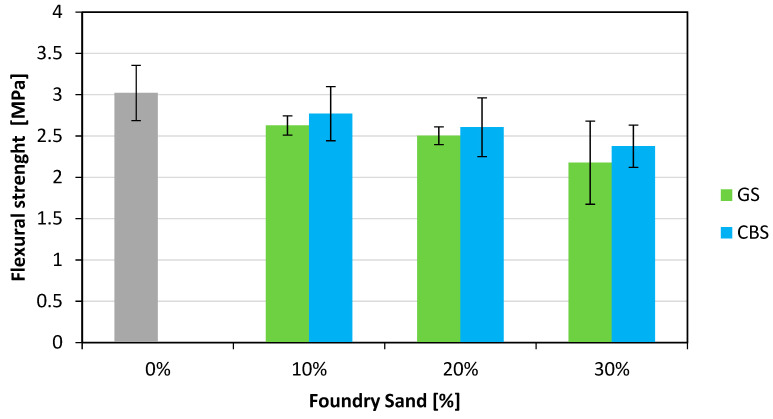
Flexural strength results.

Three-point bending tests were carried out on each specimen measuring 150 × 150 × 600 mm according to the EN 12390-5 [37] standard by using a 200 kN capacity universal testing machine (METROCOM Engineering SpA, Novara, Italy). The results are shown in Figure 10. The flexural strength shows greater reductions compared to the indirect tensile strength as the content of foundry sand increases. Specifically, for GS, the reductions are 13%, 17%, and 28% for the respective GS dosages of 10%, 20%, and 30% CBS shows reductions of 8%, 14%, and 21%.

#### 3.3.4. Water Absorption

**Figure 11:**
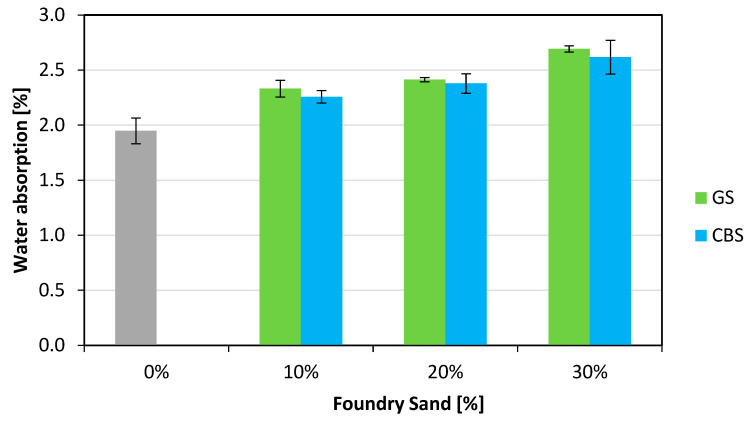
Water absorption.

The water absorption measurements of hardened concrete were carried out on cylindrical specimens with a length of 100 mm and a diameter of 100 mm, subjected to testing according to the procedures outlined in the UNI 7699 [38]. The results are shown in Figure 11. In this case, an increase in water absorption is observed with the increase in foundry sand content. Specifically, GS shows increases of 2.33%, 2.41%, and 2.69% for the respective contents of 10%, 20%, and 30%, while CBS shows slightly lower increases of 2.26%, 2.38%, and 2.62%.

**Figure 12:**
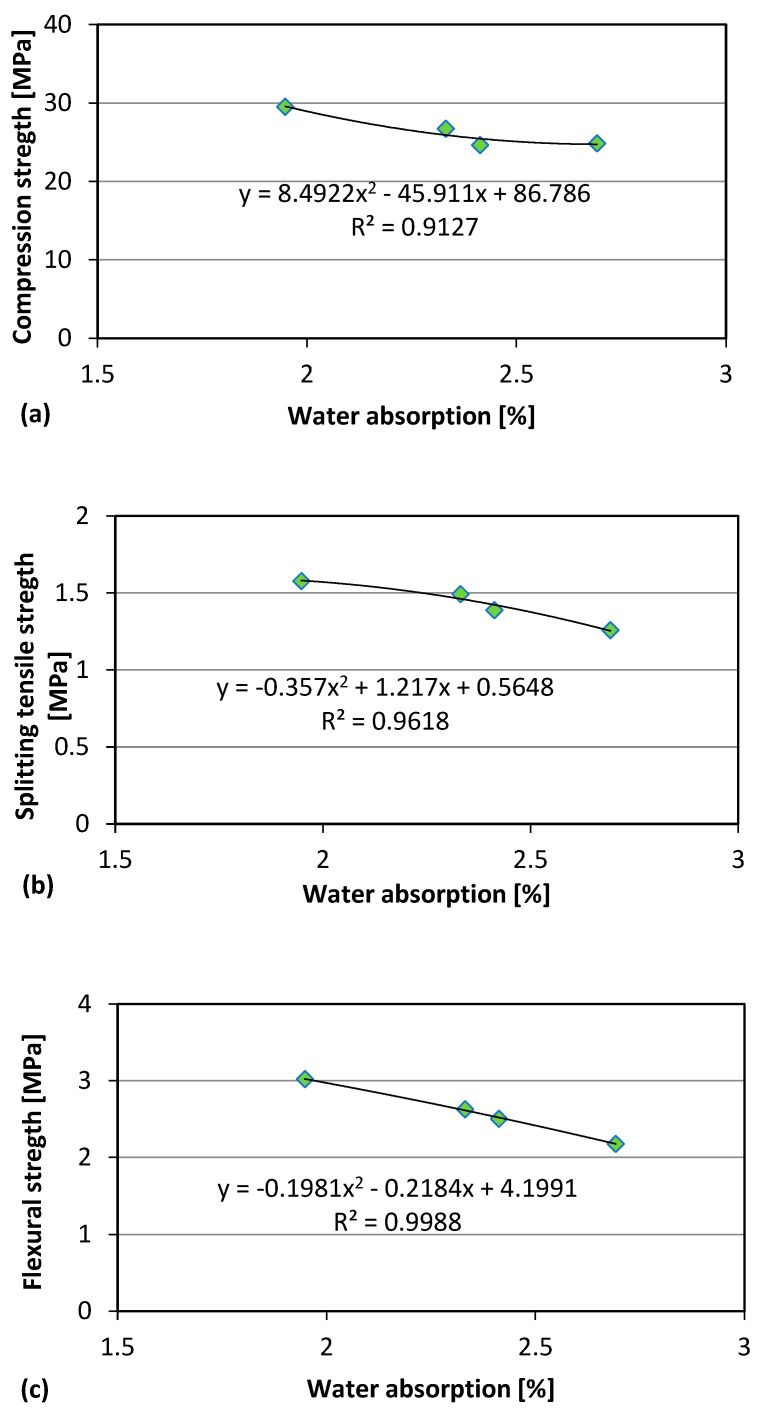
GS: Relationship water absorption-Compressive strength (**a**), water absorption-Splitting tensile strength (**b**), water absorption-Flexural strength (**c**).

**Figure 13:**
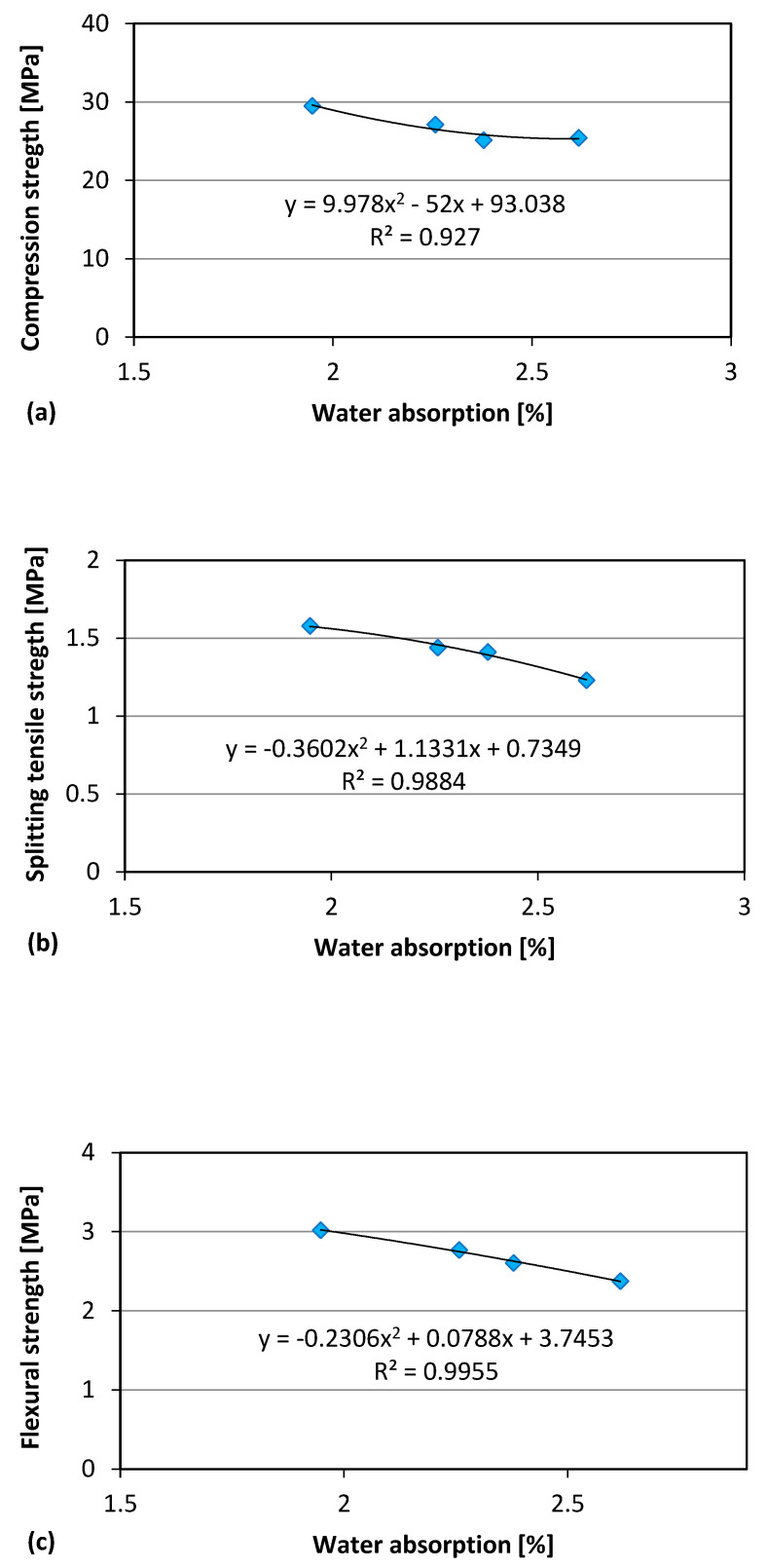
CBS: Relationship water absorption-Compressive strength (**a**), water absorption-Splitting tensile strength (**b**), water absorption-Flexural strength (**c**).

Polynomial regression analysis between the water absorption data (as indirect measure of porosity) and the mechanical properties yields the plots are presented in Figure 12 for GS and in Figure 13 for CBS. The reported experimental data show a clear correlation between the water absorption of concrete and its mechanical strength. In particular, it is observed that the strength tends to decrease as water absorption increases (indicating higher porosity). To more accurately capture this relationship, a polynomial regression was applied, enabling the modeling of the data trend with a curve that more closely fits the observed behavior. The polynomial model shows that even modest increases in porosity can lead to reductions in strength, highlighting the material’s sensitivity to its internal structure.

As a concluding summary of the results presented herein, Table 5 provides a synthesis of the mechanical strength data, normalized with respect to the reference mix, for the concrete mixtures incorporating foundry sands analyzed in this study. For comparison purposes, it is also useful to include data from a recent study by Wankhede et al. [23], which shows several similarities with the present work in terms of materials and methodologies adopted. It is worth noting that the aforementioned study investigates GSs analogous to those used here, but with significantly lower water absorption properties. This may explain why better mechanical performances are reported in [23], thereby highlighting, as stated in the Introduction, the considerable variability in the properties of foundry sands in general.

## 4. Discussion

From a chemical-physical perspective, GS is characterized by the presence of clay and carbonaceous impurities, as a consequence of the utilized binder. These impurities, in addition to being dispersed, also coat the surface of the individual grains, giving them a characteristic black color. The density of GS is comparable to that of NS, but its granulometry is much finer, with an average dimension of only 400 μm. GS display a high water-absorption rate, which is likely attributable to the presence of bentonite components. Conversely, the grains of CBS are free from dispersed impurities and exhibit a characteristic translucent appearance, presumably attributable to a uniformly distributed resin coating on the surface of the individual grains. The density of CBS is slightly higher than that of GS, but still comparable to NS. Furthermore, its granulometry appears even finer than that of GS, with an average grain size of approximately 300 μm. The water absorption of CBS is negligible, likely attributable to the presence of a resin coating that facilitates water repellency. Mixtures containing GS show a reduction in workability, requiring the incorporation of superplasticizer percentages ranging from 0.3% to 1.5% of cement content, depending on the amount of GS added. Conversely, mixtures containing CBS exhibit a workability that is comparable to that of the standard mixture ST, thereby necessitating a reduced quantity of superplasticizer. Foundry sands concrete shows a lower density, probably due to the lower density of the foundry sands (GS and CBS) compared to NS. Furthermore, the addition of foundry sands probably leads to a greater formation of voids within the matrix. During the cement hydration process, a portion of the available water is absorbed by the fine particles present in the foundry sand, thereby reducing the amount of water actually involved in the hydration reactions [1,2,3,4,5,6,7,8]. This effect is further intensified by the presence of clay binder in the sand (as seen in the ESEM images of GS), which retains an even greater quantity of water [8]. The removal of useful water can slow down or hinder cement hydration, promoting the formation of residual voids within the matrix. Specifically, the water retained by the sand creates spaces between the particles and when this water evaporates, these spaces turn into actual voids, increasing the porosity of the seasoned concrete [13].

CBS exhibits surface coatings composed of phenolic resins, substances that can also interfere with the cement hydration reaction [39]. In particular, phenolic compounds are known for their ability to delay hydration processes and increase the porosity of the material [40,41]. As specified in Section 2.2, the CBS used in the present experimental investigation originates from an aluminum foundry. Several studies [42,43] have shown that this type of material can lead to an increased pore content, with pores often exhibiting elongated shapes or forming around individual sand grains [42].

The compressive strength reductions may be attributed to the increase in porosity previously described [8,13]. As expected, the concrete specimens with reduced replacement percentages display a lower strength reduction with respect to the other ones, both for GS and CBS. A comparison of the decreasing trend at 90 days with that at 28 days reveals a high degree of similarity (Figure 7 and Figure 8) highlighting the persistence of the issue.

The strength decrease observed both in indirect tensile tests and bending tests may be attributed to the presence of binders in foundry sands, which results in the formation of a coating on the sand grains, as illustrated in Figure 3a,b and Figure 4a,b. The presence of this coating, as well as carbonaceous impurities, may have exerted deleterious effects, such as a weakening of the bond between cement and aggregate and/or a delay or reduced formation of cement hydration CSH products (Calcium Silicate Hydrated). This phenomenon may have resulted in an increase in water absorption, particularly in the case of GS with a clay coating. Conversely, for CBS characterized by a resinous coating, it may have led to a decrease in water absorption, resulting in both cases in a weakening of the transition zone at the cement/aggregate interface [8,13]. In particular, CBS appears to perform slightly better, probably because the water retention caused by the bentonite in the GS is more detrimental than the hydrophobic nature of resin-based materials. Furthermore, Figure 3 and Figure 4 show that CBS exhibits fewer dispersed impurities, which are less critical for the ITZ [12,13]. Additionally, as observed in Figure 3 and Figure 4, CBS show fewer surface asperities and a more regular morphology, resulting in a reduced tendency for the development of deformations, microcracking, and ultimately weak zones within the concrete matrix [1,12,29].

Finally, particular attention must be paid to aspects related to the durability of concrete. Water absorption is considered as an indirect measure of concrete porosity [44,45], which in turn serves as an indicator of concrete durability [4]. In the tests carried out, increases in water absorption exceeding 2% were observed in the hardened concrete for both GS and CBS mixes, accompanied by corresponding decreases in density, as previously described. As previously highlighted, the underlying causes of this increase can be attributed to the improper formation of the water–cement gel within the matrix and inadequate bonding between the aggregates and the cement, leading to the development of open pore systems [11]. On the other hand, the increased porosity resulting from the use of waste foundry sand makes concrete a lightweight, environmentally friendly and sustainable material, particularly suited to lightweight constructions [46].

In addition to causing the short-term reductions in mechanical performance observed in this study, an increase in porosity can lead to several long-term durability issues [1,2,3,4,5,6,7]. A more porous structure allows water and aggressive agents such as chlorides, sulfates, and acids, to penetrate the cementitious matrix more easily, thereby accelerating degradation processes. In cold climates, water retained in the pores may freeze and expand in volume, generating microcracks that further deteriorate concrete performance over time. Moreover, increased air permeability accelerates carbonation processes, which have additional detrimental effects on durability.

In conclusion of the present section, it is important to highlight as a novel element that, while scientific literature already offers a substantial amount of experimental data on GS, the available information on CBS remains very limited [12]. Further experimental investigations are needed to provide a sufficiently comprehensive overview of CBS, comparable to that of GS. This study is therefore positioned as a contribution in this direction.

## 5. Conclusions

From the current study, the following conclusions can be derived:

1. The properties of fresh concrete decay in terms of workability as the content of foundry sand increases, particularly in mixtures containing GS, which requires greater use of superplasticizers due to its elevated water absorption caused by bentonite.
2. In the hardened state, concrete with foundry sands exhibits lower density compared to the reference mixture. This reduction is primarily attributed to the fine granulometry and high specific surface area of the sands, which increase water demand during mixing and hinder proper crystal formation during cement hydration.
3. The compressive strength of the concrete decreases with increasing foundry sand content. Although CBS shows slightly better performance than GS, both materials display similar overall trends. The extension of the curing time from 28 to 90 days has been demonstrated to enhance strength. However, the rate of strength reduction remains consistent, indicating a persistent influence of porosity.
4. The presence of coatings in foundry sands, even if of different nature in GS and CBS, has a detrimental effect on the adhesion between cement and aggregate, since it weakens the interfacial zones and causes a reduction both in tensile and flexural strength, as the content of foundry sand increases.
5. Hardened concretes containing GS and CBSs exhibited higher water absorption. This, confirming increased porosity, may pose a durability concern for concrete exposed to aggressive environments.

In summary, it can be concluded that the use of foundry sands, both for GS and CBS, generally lowers the physical–mechanical performance of concrete. However, this occurrence should not be regarded as a substantial impediment, given that a 10% replacement rate resulted in compressive strength reductions of 9% and 8% for GS and CBS, respectively. For a 10% replacement rate of foundry sand, a reduction of less than 10% could be considered acceptable for non-structural concrete applications, including screeds, pavements, and light precast elements [17,46]. Alternatively, lower replacement rates, below 10%, could also be considered, in line with the 2022 decree issued by the Italian Ministry of Ecological Transition [47], which mandates that concrete must contain at least 5% recycled or recovered materials. Finally, the performance reductions may be mitigated through the modification of the foundry sand characteristics, in particular, by eliminating the very fine granulometric fraction present in both GS and CBS. Furthermore, the implementation of mechanical and/or chemical treatments aiming to eliminate or reduce the clayey/carbonaceous impurities present in GS could also contribute to achieving this objective.

## 6. Limitations

Despite the promising results obtained, this study presents several limitations that should be mentioned. No tests were conducted for replacement rates below 10%, which could have provided more precise insights into the applicability of minimum recycled content requirements. Moreover, specific durability assessments, such as exposure to chloride attack or freeze–thaw cycles, were not carried out. The study also did not investigate the leaching behavior or the potential environmental impacts associated with the reuse of CBS, which contains phenolic resins.

## Figures and Tables

**Table 1:** Reference Concrete mix proportions.

Cement	350 kg
Water	150 kg
Water cement ratio	0.43
Fine aggregate (0–4 mm)	592 kg
Coarse aggregate (4–16 mm)	1240 kg

**Table 2:** Natural sand (NS) composition.

Constituent Oxide	[wt%]
SiO_2_	79.8%
Fe_2_O_3_	2.42%
Al_2_O_3_	3.28%
CaO	4.60%
MgO	2.18%
Na_2_O	2.00%
K_2_O	1.96%

**Table 3:** Mixture proportions of concretes containing GS and CBS.

Mixture	Cement [kg]	Water [kg]	Gravel [kg]	NS [kg]	GS [kg]	CBS [kg]	Superplasticizer [%c]
ST-0%	350	150	1240	592	0	0	0
GS-10%	350	150	1240	532.8	59.2	0	0.3
GS-20%	350	150	1240	473.6	118.4	0	0.7
GS-30%	350	150	1240	414.4	177.6	0	1.5
CBS-10%	350	150	1240	532.8	0	59.2	0.2
CBS-20%	350	150	1240	473.6	0	118.4	0.2
CBS-30%	350	150	1240	414.4	0	177.6	0.2

**Table 4:** Chemical composition of GS and bentonite.

Constituent Oxide	NS [wt%]	Bentonite [wt%]	GS [wt%]
Na_2_O	2.00	2.7 ± 0.2	1.3 ± 0.7
MgO	2.18	4.1 ± 0.2	0.9 ± 0.4
Al_2_O_3_	3.28	20.2 ± 0.4	9.1 ± 0.4
SiO_2_	79.80	62.1 ± 0.8	83.4 ± 2
SO_3_	-	1.0 ± 0.1	0.7 ± 0.4
K_2_O	1.96	0.7 ± 0.1	0.4 ± 0.3
CaO	4.60	1.9 ± 0.2	0.8 ± 0.3
TiO_2_	-	0.5 ± 0.2	0.2 ± 0.1
Fe_2_O_3_	2.42	4.1 ± 0.4	3.3 ± 0.6

**Table 5:** Normalized mechanical strength data compared with a recent study [23].

Mixture	Compression Strength [f_%_/f_ST_]	Splitting Tensile Strength [f_%_/f_ST_]	Flexural Strength [f_%_/f_ST_]
ST-0%	1.00	1.00	1.00
GS-10%	0.90	0.95	0.87
GS-20%	0.83	0.88	0.83
GS-30%	0.84	0.80	0.72
CBS-10%	0.92	0.91	0.92
CBS-20%	0.85	0.89	0.86
CBS-30%	0.86	0.78	0.79
[23]-10%	0.94	1.08	1.03
[23]-20%	1.02	1.13	1.10
[23]-30%	0.93	1.11	1.00

## Data Availability

The original contributions presented in this study are included in the article. Further inquiries can be directed to the corresponding author.
